# The Impact of SARS-CoV-2 on Patients With Lower Urinary Tract Symptoms (LUTS)

**DOI:** 10.7759/cureus.59148

**Published:** 2024-04-27

**Authors:** Marian S Balacescu, Cosmin V Ene, Dragos Eugen Georgescu, Catalin A Bulai, Adrian Militaru, Corina D Ene, Ileana Adela Vacaroiu, Dragos A Georgescu, Bogdan F Geavlete, Petrisor Geavlete

**Affiliations:** 1 Urology, Carol Davila University of Medicine and Pharmacy, Bucharest, ROU; 2 Urology, "Sf. Ioan" Clinical Emergency Hospital, Bucharest, ROU; 3 General Surgery, Carol Davila University of Medicine and Pharmacy, Bucharest, ROU; 4 General Surgery, Dr. I. Cantacuzino Clinical Hospital, Bucharest, ROU; 5 Nephrology, Carol Davila University of Medicine and Pharmacy, Bucharest, ROU; 6 Nephrology, "Dr. Carol Davila" Clinical Nephrology Hospital, Bucharest, ROU; 7 Nephrology, "Sf. Ioan" Clinical Emergency Hospital, Bucharest, ROU

**Keywords:** healthcare outcomes, turp, luts, bph management, sars-cov2

## Abstract

Introduction: During the severe acute respiratory syndrome coronavirus 2 (SARS-CoV-2) pandemic, the management of patients with lower urinary tract symptoms (LUTS) underwent dynamic adjustments in response to an evolving understanding of the virus's impact on different patient populations. Healthcare practitioners reevaluated therapeutic approaches for conditions like benign prostatic hyperplasia (BPH), considering the potential implications of this condition on the severity and progression of coronavirus disease 2019 (COVID-19). This study aims to investigate potential correlations between SARS-CoV-2 infection severity, exacerbation of LUTS, and BPH progression.

Material and methods: This retrospective study includes patients hospitalized in our Urology Department between January 2021 and January 2023, presenting with both SARS-CoV-2 and BPH. Their ages ranged from 57 to 88 years, with a mean age of 65.4 years. The diagnosis of BPH relied on a diagnostic triad consisting of digital rectal examination, biological markers (including prostate-specific antigen (PSA) and free PSA, and ultrasound examination, with both conditions confirmed based on test results. Transurethral resection of the prostate (TURP) procedures utilized monopolar Karl Storz resection equipment, using sorbitol and bipolar Olympus devices for transurethral resection of the prostate in saline (TURPis). Haemostasia was performed using roller balls. Anticoagulation followed a prescribed scheme by cardiologists and infectious disease specialists. Statistical analysis was conducted using IBM Corp. Released 2013. IBM SPSS Statistics for Windows, Version 22.0. Armonk, NY: IBM Corp.

Results: Among the 138 hospitalized patients affected by both BPH and COVID-19, 18 required emergency endoscopic procedures (specifically TURP or TURPis) to achieve hemostasis (Figures 1, 2). These individuals presented persistent hematuria despite conservative treatments. The mean duration of surgery was 57.9 minutes. Patients who underwent surgery had a longer average hospital stay compared to those who did not, with durations of 10.5 days versus 7.5 days, respectively. Additionally, urethrovesical catheter insertion was necessary in 29 cases due to acute urinary retention or worsening voiding symptoms during hospitalization. These patients are scheduled for further urological evaluation following the resolution of the COVID-19 episode. In a cohort of 53 patients for whom data were accessible, comparisons were made between the pre-COVID status and the levels of the International Prostate Symptom Score (IPSS), post-voiding residue (PVR), and quality of life (QoL). The findings revealed a mean pre-COVID IPSS value of 11.6 and a COVID-related value of 14.2, with a statistically significant difference noted (p < 0.05). The mean pre-COVID PVR was 42.3 cm^2^, whereas during the COVID-19 period, it measured 62.5 cm^2^, also exhibiting a significant difference (p < 0.05). Additionally, the QoL showed a mean pre-COVID-19 score of 2.4 and a COVID-19-associated score of 2.9, again demonstrating statistical significance (p < 0.05).

Conclusion: The onset of the SARS-CoV-2 pandemic posed novel challenges in the medical realm, impacting the approach to BPH management. A common practice was delaying treatment for chronic BPH until viral infection remission to reduce associated risks. Additionally, our study revealed a worse evolution in LUTS among individuals with severe COVID-19 symptoms.

## Introduction

The emergence of the ongoing worldwide pandemic of coronavirus disease 2019 (COVID-19), triggered by the severe acute respiratory syndrome coronavirus 2 (SARS-CoV-2), initially detected in late 2019 in Wuhan, China, resulted in an immense global crisis [1]. Throughout this timeframe, our approach to handling patients presenting both with BPH and COVID-19 aimed to address the challenges stemming from the viral infection while ensuring that the management of BPH did not significantly exacerbate the viral pathology. Consequently, it is plausible to infer that a considerable portion of older male patients, particularly those diagnosed with severe cases of COVID-19, may concurrently experience BPH. Additionally, the viral infection might intensify LUTS in these individuals. Recent studies have suggested that LUTS might exacerbate the early manifestations of COVID-19 and that the International Prostate Symptom Score (IPSS), particularly in older males, maybe a possible complication of this disease [2,3]. The suggested link between metabolic variables and SARS-CoV-2 infection seems oversimplified. Metabolic syndrome (MetS) is a collection of metabolic disorders linked to central obesity and insulin resistance that are linked to a higher risk of cardiovascular illnesses [4]. Insulin resistance, increased visceral adiposity, changes in sex hormones, and cellular inflammatory responses significantly contribute to the link between metabolic syndrome and prostatic illnesses, even though the precise molecular mechanism is still unclear [5]. Nevertheless, the potential association between LUTS and SARS-CoV-2 infection presents an even more intricate scenario. LUTS, characterized by a multifactorial etiology, manifests in both genders with comparable prevalence [6]. There is a small study by Kaya and colleagues that recorded a statistically significant difference of less than 1 point in IPSS storage score in patients after SARS-CoV-2 infection with no difference in voiding or total IPSS [2].

The emergence of SARS-CoV-2 has prompted alterations in the management approach for individuals with BPH, resulting in a sequence of modifications in the trajectory of case evolution. Notably, in urgent scenarios such as acute urinary retention or total hematuria, there has emerged a preference among both patients and medical practitioners for minimal interventions and deliberate timing for surgical interventions. This preference corresponded with decreased operative durations and reduced average hospitalization periods, particularly observed in non-COVID-19 patients [7].

## Materials and methods

The study is retrospective and includes the patients who presented with SARS-CoV-2 and BPH and who were hospitalized in our Urology Department between January 2021 and January 2023. The age range of the patients spanned from 57 to 88 years, with a calculated mean age of 65.4 years. The diagnostic triad utilized for the diagnosis of BPH comprised the digital rectal examination, biological markers (PSA, free PSA), and ultrasound assessment. At the time of their admission, some patients admitted to the hospital were already receiving treatment with alpha-blockers and/or 5-alpha-reductase inhibitors, a fact that is worth mentioning.

Our inclusion criteria were: (1) hospitalized patients; (2) patients with BPH and normal total prostate-specific antigen (TPSA) and free PSA/total PSA (f/tPSA) ratios before admission; (3) patients with prostate nodules that were observed under color Doppler ultrasonography, computed tomography (CT), magnetic resonance imaging (MRI), or physical examination, but whose prostate biopsy confirmed BPH before admission; (4) patients with a TPSA > 10 ng/ml or a TPSA at 4-10 ng/ml but abnormal f/tPSA and prostate-specific antigen density (PSAD) in whom prostate cancer was excluded by previous prostate biopsy before admission and (5) patients diagnosed with SARS-CoV-2 using reverse transcription polymerase chain reaction (RT-PCR), admitted in our department for low urinary tract symptoms.

Exclusion criteria were: (1) patients with prostate nodules as ascertained under color Doppler ultrasonography, CT, MRI, or physical examination, in whom no prostate biopsy was performed to rule out prostate cancer, and (3) BPH patients with a TPSA > 10 ng/ml or a TPSA at 4-10 ng/ml but with abnormal f/tPSA and PSAD, in whom prostate cancer was not eliminated.

The admission procedure was carried out on every patient, which included skeletal scintigraphy, a thoracic CT scan, an electrocardiogram, general ultrasonography, urine tests (including urine culture and urine summary), and blood tests (hemogram, glycemia, urea, creatinine, coagulation test, ferritin, D-dimers, aspartate aminotransferase (GOT), alanine aminotransferase (GPT), total and direct bilirubin, C-reactive protein (CRP), PSA, free PSA. In specific cases, an additional CT scan of the abdomen and pelvis was performed. During the procedures, monopolar Karl Storz resection equipment was utilized for the TURP, whereas a bipolar Olympus device was utilized for the TURPis. In both techniques, hemostasis was performed using roller balls. All the patients were anticoagulated according to a scheme prescribed by a cardiologist and infectious diseases doctor.

The qualitative data were represented as proportions and percentages. The mean and standard deviation (SD) of quantitative data were analyzed using the chi-square test. P-values less than 0.05 were regarded as statistically significant. IBM Corp. Released 2013. IBM SPSS Statistics for Windows, Version 22.0. Armonk, NY: IBM Corp. was used to examine the data.

## Results

Of the studied patients, there were 138 cases with associated comorbidities besides SARS-CoV-2 infection: 86 cases with cardiac pathology (arterial hypertension (AHT), chronic cardiac disease (angina), 23 cases with metabolic disease (obesity, diabetes mellitus), 17 cases with chronic kidney disease (CKD), 23 cases with infectious disease (Hepatitis B virus (HBV), Hepatitis C virus (HCV), 15 cases with a history of neoplasia (colonic or rectal neoplasia), and five cases with chronic respiratory diseases (BPOC) (Table 1). Hence, a multidisciplinary approach was pursued to ensure the delivery of optimal personalized treatment for the patients, encompassing expertise from cardiovascular, nephrology, pulmonology, and gastroenterology specialties. Moreover, individuals with a history of colonic neoplasia underwent a meticulous examination of specific systemic inflammatory markers, such as neutrophil/lymphocyte ratio (NLR), platelet/lymphocyte ratio (PLR), and lymphocyte/monocyte ratio (LMR), to assess the prognosis of their condition. All patients exhibited results within the normal range.

**Table 1:** Associated comorbidities in patients with benign prostatic hyperplasia (BPH) associated with SARS-CoV-2. AHT: Abusive head trauma, CKD: Chronic kidney disease, VHB: Hepatitis B virus, VHC: Hepatitis C virus, COPD: Chronic obstructive pulmonary disease

Associated pathology	Number of cases
Cardiac pathologies (AHT, chronic cardiac disease, angina)	86
Metabolic disease (obesity, diabetes mellitus)	23
Chronic kidney disease (CKD)	17
Infectious diseases (VHB, VHC)	23
History of neoplasia (colonic or rectal neoplasia)	15
Chronic respiratory diseases (COPD)	5

Regarding the severity assessment of the SARS-CoV-2 infection, evaluation primarily involved thoracic CT scans delineating pulmonary damage. Among the cases examined, pulmonary damage ranged from 0-30% in 59 instances, 30-70% in 43 cases, and 70-99% in 36 cases. It could be observed from each group that the most pulmonary aggressive subtype in our group was the COVID-19 subtype Delta, with the most significant number of severe or potentially severe cases. Apart from respiratory complications, no other form of urological complication associated with SARS-CoV-2 pathology was observed within this group.

**Figure 1:**
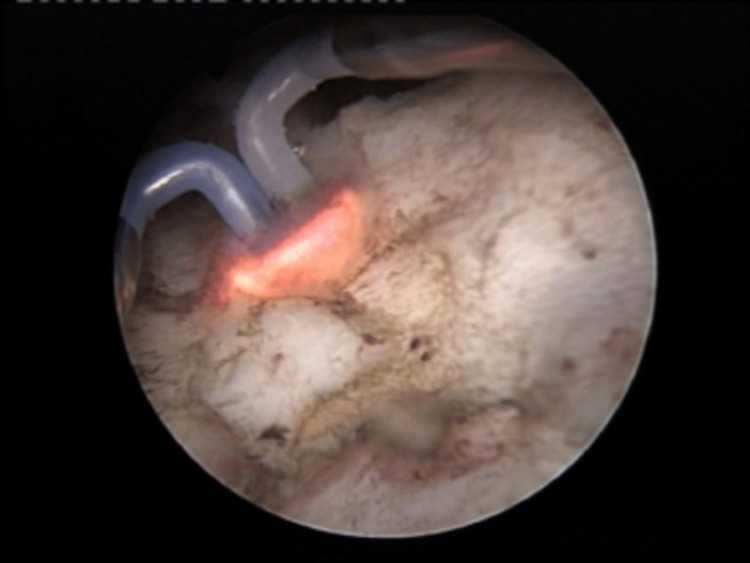
Benign prostatic hyperplasia (BPH) in COVID-19 patients. Bipolar hemostasis after resection.

**Figure 2:**
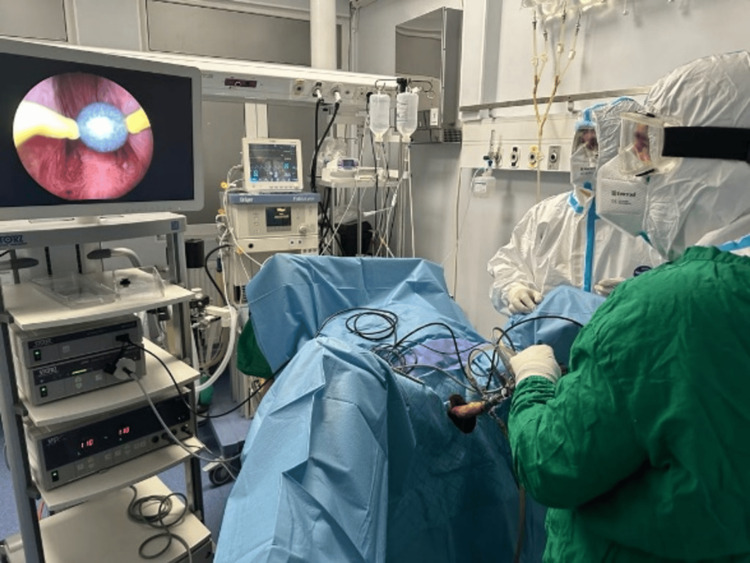
Intraoperative image during the COVID-19 period.

Out of the 138 hospitalized cases, 18 required an urgent endoscopic procedure under spinal anesthesia (TURP or TURPis) for hemostasis (Figures 1, 2). These patients had chronic hematuria that had not improved with conservative care. The average operatory time was 57.9 minutes, and the average hospitalization period was higher in operated patients than in non-operated cases, at 10.5 days versus 7.5 days.

Twenty-nine cases required a urethrovesical catheter due to acute urinary retention or worsening voiding symptoms during hospitalization. Subsequently, these patients are scheduled for further urological evaluations after the COVID-19 episode. Of the remaining cases of BPH, 27 started receiving alpha-blocker treatment while they were hospitalized along with 5 alpha-reductase inhibitors, whereas in 74 cases, no urological interventions were undertaken to alter the treatment protocol and management solely centered on addressing the COVID-19 infection. Among these cases, 25 already had chronic urinary retention at the time of admission and had a urethrovesical probe present. Except for a group of 39 instances that required ventilatory support, of whom 13 cases died, the cases' evolution was positive, with low oxygen needs (less than 5 l/h) in 49 cases and discharged without complications. Eleven patients necessitating emergent endoscopic intervention also required ventilatory support, resulting in an extended hospital stay compared to other cases requiring ventilatory assistance (averaging 21.3 days versus 15.2 days). The IPSS level, PVR, and QoL were compared with the pre-COVID-19 status in a group of 53 patients where these data were available. Thus, we observed a mean IPSS pre-COVID-19 value of 11.6 and a COVID-19 value of 14.2, p<0.05; for PVR, the mean pre-COVID-19 value was 42.3 CMC, while the mean COVID value was 62.5 CMC, p<0.05. The QoL also had a mean pre-COVID-19 value of 2.4 and a COVID-19 value of 2.9, p<0.05 (Table 2). 

**Table 2:** International prostate symptom score (IPSS), post-voiding residue (PVR), and quality of life (QoL) values before and during SARS-CoV-2 infection.

	Pre-COVID value	COVID value	p-value
IPSS	11.6	14.2	<0.05
PVR	42.3 cmc	62.5 cmc	<0.05
QoL	2.4	2.9	<0.05

The PSA value increased in the more severe SARS-CoV-2 cases in correlation with CRP and with the pulmonary damage (Table 3): for the cases with 0-30% pulmonary damage, CRP (mg/dL) was 60.2 ±17.4 and PSA increase rate (ng/dL) was 0.84±0.32, for 30-70% pulmonary damage, CRP (mg/dL) was 83.2±21.4 and PSA increase rate (ng/dL) was 1.23±0.51, while for 70-99% pulmonary damage, CRP (mg/dL) was 119.1±29.3 and PSA increase rate (ng/dL) was 1.79±0.86, while p-values were <0.05 for all the compared parameters. The PSA increase was calculated by making a difference between the initial PSA (measured by the patient before SARS-CoV-2) and the PSA at admission to the hospital.

**Table 3:** The prostate specific antigen (PSA) increase and the C-reactive protein (CRP) value correlated with the respiratory damage subtype.

	0-30% pulmonary damage	30-70% pulmonary damage	70-99% pulmonary damage	p-value
CRP (mg/dL)	60.2 ±17.4	83.2±21.4	119.1±29.3	<0.05
PSA increase (ng/dL)	0.84±0.32	1.23±0.51	1.79±0.86	<0.05

## Discussion

BPH is recognized as a general risk factor associated with a spectrum of medical conditions, linked to systemic consequences and comorbidities. Research suggests that BPH may predispose individuals to cardiovascular diseases, metabolic disorders, and musculoskeletal issues such as osteoarthritis. First, BPH-related hormonal imbalances, particularly alterations in testosterone and estrogen levels, may contribute to bone remodeling and metabolism, potentially influencing the development of benign cavitary bone lesions (BCBL). While emerging treatment modalities for BCBL, such as tricalcium phosphate and hydroxyapatite, have shown promise in promoting bone regeneration and healing, their potential role warrants consideration in the comprehensive management approach for patients presenting with both BPH and BCBL [8].

Second, BPH has been implicated in increasing the risk of certain surgical interventions, including total hip arthroplasty (THA) and total knee arthroplasty (TKA), likely due to its association with aging and chronic inflammation [9,10]. Understanding the broader impact of BPH beyond urological concerns is crucial for comprehensive patient management and preventive strategies. Third, BPH has garnered attention in orthopedic practice due to its potential influence on radiological digital planning accuracy for THA. While recent studies have underscored the importance of precise preoperative planning in THA to achieve optimal surgical outcomes, the presence of BPH, often necessitating urinary catheterization or pharmacological management, can introduce complexities in patient positioning and imaging acquisition during preoperative planning [11].

The COVID-19 virus had a substantial impact across global surgical disciplines shortly after the onset of the pandemic. A study from Italy mentions that two-thirds of the hospital beds were occupied by COVID-19 patients. Within 15 days, there was a 30% reduction in urological surgeries, leading to the complete closure of the facility on March 19, 2020. The scarcity of anesthesiologists, ventilators, and operating rooms for critically ill patients has limited their capacity to perform certain urgent urological surgeries [12].

Recent research has shown that COVID-19 has widespread impacts on numerous organs. The first potential finding of COVID-19 as one of the impacts above was the rise in LUTS [2]. Although angiotensin-converting enzyme 2 (ACE 2) expression is highest in the lungs, intestines, and kidneys, it is also higher in 2.4% of urothelial cells, which makes individuals more vulnerable to COVID-19-caused viral cystitis. Through another pathophysiological pathway, the inflammatory conditions that develop because of viral cystitis or bacterial infection may exacerbate the symptoms of LUTS [3].

The progression of BPH, however, maybe accelerated by prostatic inflammation, according to some research [13]. Activation of pro-inflammatory pathways, increased cytokine production, and consequently inflammatory reactions in susceptible organs, including the prostate, have all been linked to infection with SARS-CoV-2 and subsequent reduction of ACE2 [14]. Therefore, SARS-CoV-2 may result in irritable symptoms and start an inflammatory response in the prostate gland [15]. Thus, the observed correlation in our study between the progression of BPH symptoms and parameters could potentially be linked to pulmonary damage induced by heightened inflammatory conditions [16]. The higher prevalence of the inflammatory syndrome observed in operated cases, in contrast to those not subjected to surgery, may contribute to the escalated morbidity rate in patients who underwent the procedure. Additionally, our study noted a higher percentage of complications in the operated group compared to the non-operated cohort. This discrepancy between the rates of complications could potentially explain the elevated morbidity rate among those who underwent surgery. However, no research has been done so far to examine the probability of BPH development as a side effect of SARS-CoV-2 infection.

Literature studies have proposed a correlation between BPH and COVID-19, suggesting a connection to worsening voiding symptoms during a COVID-19 infection. These propositions entail four theories: renin-angiotensin system (RAS) dysregulation [17], the androgen-related theory postulating an exacerbation of BPH symptoms due to increased androgen receptor activity associated with elevated dydrogesterone [18], inflammation-related factors [19], and metabolic derangement associations [20]. The worsening of voiding symptoms and the increase of CRP were also observed in our patients, especially in correlation with an increased pulmonary damage percentage, so we intuitively tried to decrease the inflammatory status. In a quite similar number of patients (50 patients) with BPH and COVID-19, Nabeeh et al. found that IPSS significantly increased during the hospital stay as well as one and three months after discharge (26.6 5.8, 25.4 5.86, and 25.1 6.3), compared to IPSS before infection (13.4 4.3, p 0.001). The authors also found a substantial decline in quality of life (QoL) from COVID-19's pre-illness value of 3.4 1.0 to 5.3 0.7 (p 0.001) following recovery [21], comparable to our outcomes. Also, recent growing research has suggested that IPSS, particularly in older males, maybe a potential consequence of this disease and that LUTS may enhance early symptoms of COVID-19 [2,22].

In recent years, a plethora of investigations have been initiated, elucidating the association between systemic inflammatory response and malignancies. As a result, individuals with a history of neoplasia underwent a meticulous examination of specific markers such as NLR, PLR, and LMR to assess the prognosis of their condition. According to the specialized literature, NLR and platelet-to-lymphocyte ratio PLR are identified as risk factors, whereas LMR is regarded as a protective factor for survival [23].

Moreover, the coexistence of BPH and colon cancer poses complexities in decision-making regarding the most appropriate surgical approach. Factors such as the patient's overall health, the stage of colon cancer, and the extent of BPH-related symptoms must be meticulously considered to ensure optimal treatment outcomes. Challenges may arise in balancing the surgical interventions required for both conditions, particularly when selecting the appropriate timing and sequence of procedures. Additionally, the potential impact of BPH on perioperative care and postoperative recovery necessitates a comprehensive and multidisciplinary approach. Addressing these challenges involves collaborative efforts between urologists, colorectal surgeons, and other specialists to tailor surgical strategies that prioritize patient safety and overall well-being [24].

An intriguing aspect of our study was the observation that the blood PSA levels during the active phase of the disease exhibited a statistically significant elevation compared to the levels measured both before and after the disease onset. This study undertook a comparative analysis, aligning its findings with those documented in specialized literature. The focus of the investigation was the assessment of blood PSA levels in male patients diagnosed with COVID-19, with measurements taken both before the onset of COVID-19 and during the active infection period. Notably, the outcomes demonstrated a concurrence with previous research, revealing similarities across 91 cases in the context of PSA levels [25]. Overall, it could be observed that the same inflammatory route may contribute to the worsening of BPH-related LUTS and associated consequences both during and after infection with SARS-CoV-2 [26].

## Conclusions

The SARS-CoV-2 pandemic generated new challenges for all the medical fields, including the urological pathologies, including BPH, where the general management trend was to delay chronic cases until the time of remission of viral infection to reduce the morbidity as much as possible. Also, it was observed that the patients with severe forms of COVID-19 had a worse evolution of the LUTS too. Additional elucidation is anticipated regarding the precise mechanisms underlying the associated pathologies mentioned in this study.
